# THE INTERPLAY OF FUNCTIONAL PERFORMANCE AND COGNITION ON WALKING SPEED RESERVE

**DOI:** 10.1093/geroni/igz038.606

**Published:** 2019-11-08

**Authors:** Nicole T Dawson, Ashleigh Trapuzzano

**Affiliations:** University of Central Florida, Orlando, Florida, United States

## Abstract

Domains of functional performance (strength, balance) and cognition (executive function, processing speed) have been shown to independently predict both comfortable and gait speed. Walking speed reserve (WSR) reflects an individual’s ability to increase their walking speed when needed to adjust to environmental demands. Increasing speed requires a higher demand of neuromuscular control and cognitive resources. Few studies have investigated WSR; however, poorer cognitive status has been shown to be associated with a smaller WSR. Understanding mechanisms in WSR could help with intervention development. The purpose of this study was to investigate the interplay of functional performance and cognition on WSR. Sixty-seven community-dwelling older adults (mean age 80.57; 71% female) completed assessments including: global cognition (Mini Mental State Examination), leg strength (30 Second Chair Stand), balance (Functional Reach), executive function (Trail Making Test Part-B, clock-drawing test, Flanker Task), simple reaction time, processing speed (Digit Symbol Substitution Test), and gait speed (comfortable and fast) using the GAITRite® system. WSR (fast-comfortable gait speed) was the dependent variable. Independent variables in the regression model were selected using Pearson correlation values <0.05 (strength (r=.332, p=.006) and processing speed (r=.361, p=.003)). Linear regression revealed that the strength and processing speed explained 16.3% of the variance (adjusted r2) of WSR and were both unique predictors of WSR indicating individuals with greater strength (B=.79, p=0.036) and faster processing speed (B=.44, p=0.015) have a greater capacity to alter their gait speed. Further, the interdependence between cognition and physical performance emphasizes the importance of interprofessional care for older adults.

